# Bangladesh: a success case in combating childhood diarrhoea

**DOI:** 10.7189/jogh.09.020803

**Published:** 2019-12

**Authors:** Sk Masum Billah, Shahreen Raihana, Nazia Binte Ali, Afrin Iqbal, Mohammad Masudur Rahman, Abdullah Nurus Salam Khan, Farhana Karim, Mohd Anisul Karim, Aniqa Hassan, Bianca Jackson, Neff Walker, M Altaf Hossain, Sukumar Sarker, Robert E Black, Shams El Arifeen

**Affiliations:** 1 Maternal and Child Health Division, International Centre for Diarrhoeal Disease Research, Bangladesh (icddr,b), Dhaka, Bangladesh; 2 Johns Hopkins University, Bloomberg School of Public Health, Department of International Health, Institute for International Programs, Baltimore, Maryland, United States; 3 Directorate General for Health Services, Government of Bangladesh, Dhaka, Bangladesh; 4 USAID Bangladesh, Dhaka, Bangladesh

## Abstract

**Background:**

Bangladesh had a large reduction in childhood deaths due to diarrhoeal disease in recent decades. This paper explores the preventive, promotive, curative and contextual drivers that helped Bangladesh achieve this exemplary success.

**Methods:**

Primary and secondary data collection approaches were used to document trends in reduction of Diarrhoea Specific Mortality Rate (DSMR) between 1980 and 2015, understand what policies and programmes played key roles, and estimate the contribution of specific interventions that were implemented during the period. Data acquisition involved relevant document reviews and in-depth interviews with key stake-holders. A systematic search of literature was undertaken to explore socio-economic, aetiological, behavioural, and nutritional drivers of diarrhoeal disease reduction in Bangladesh. Finally, we used LiST (Lives Saved Tool) to model the contributions of the relevant interventions during three time periods (1980-2015, 1980-2000 and 2000-2015), and to project the number of lives saved in 2030 (compared to 2015) if these interventions were implemented at near universal coverage (90%).

**Results:**

The factors which likely had the most impact on DSMR were the coordinated efforts of the Government of Bangladesh (GoB) with non-government organizations (NGOs) and the private sector that enabled swift implementation, at scale, of interventions like oral rehydration solution (ORS) and zinc, promotion of breastfeeding, handwashing and sanitary latrines (WASH), as well as improvements in female education and nutrition. Compared to 1980, we found ORS and reduction in stunting prevalence had the greatest impact on DSMR, saving roughly 70 000 lives combined in 2015. Until 2000, ORS had a higher contribution to DSMR reduction than reduction in stunting prevalence. This proportionate contribution was reversed during 2000-2015. At near universal coverage (90%) of combined direct diarrhoeal disease, nutrition and WASH interventions, we project that an additional 5356 deaths due to diarrhoea could be averted in 2030.

**Conclusion:**

Bangladesh’s achievement in reduction of DSMR highlights the important role of an enabling policy environment that fostered coordinated efforts of the public and private sectors and NGOs for maximal impact. To maintain this momentum, evidence-based interventions should be scaled up at universal coverage.

Diarrhoeal disease remains a leading global cause of childhood deaths despite a decline in the last three decades [1]. Developing countries make the largest contribution to the global burden of diarrhoea mortality with most deaths occurring in sub-Saharan Africa and South Asia. Diarrhoea has been one of the major causes of child mortality in Bangladesh, but has declined recently [1]. The explanation for this impressive reduction of diarrhoea-related childhood mortality does not lie in any one factor and would require that we critically examine the socioeconomic developments, relevant policy changes, shifts in health systems, and changes in programmes and interventions in Bangladesh which address the preventive, promotive, and curative practices for diarrhoeal disease management [2-5].

This report explores how Bangladesh was able to achieve this large reduction in childhood diarrhoea deaths over the past several decades despite being a resource-constrained densely populated country. We explored the trends in under-five diarrhoeal mortality and morbidity rates and have conducted an in-depth analysis of the complex set of factors – from changes in aetiology to contextual drivers that may have contributed to this reduction between 1980 and 2015. The analysis presented in this paper adds a unique South Asian perspective to the global understanding of this important public health problem and would help identify the critical inputs necessary to revive the global childhood diarrhoea prevention and control efforts. The analysis is also important as Bangladesh is suffering from the effects of its success, with considerable complacency about diarrhoea and a perception that this is a problem solved.

## METHODS

Primary and secondary data collection approaches were used to document reduction of Diarrhoea Specific Mortality Rate (DSMR) between 1980 and 2015, understand what policies and programmes played key roles, and estimate the contribution of specific interventions that were implemented during the period. Data acquisition involved relevant document reviews and in-depth interviews with key stake-holders. A systematic search of literature was undertaken to explore socio-economic, aetiological, behavioural, and nutritional drivers of diarrhoeal disease reduction in Bangladesh. Finally, we used the Lives Saved Tool (*LiST*) – a child survival modelling tool [6] to identify the contribution of different factors in reduction of under-five Diarrhoea Specific Mortality Rate (DSMR) between 1980 and 2015.

### Data sources

To describe trends of childhood diarrhoea mortality, we extracted national estimates of all-cause and diarrhoeal disease related mortality from different sources including the Maternal and Child Epidemiology Group working with the World Health Organization and the United Nations Interagency Group on Mortality Estimation, from 1980 to 2015. We also used data from six Bangladesh Demographic and Health Surveys (BDHS) to assess trends in coverage of therapeutic management of diarrhoeal disease (ORS, zinc, and antibiotics), changes in nutritional status indicators (stunting and wasting) and coverage of other interventions (WASH, vitamin A supplementation, breastfeeding, preventive zinc, and vaccination) [7-13].

Information on policy and programmatic changes in diarrhoea management were obtained from interviews of six key informants, all based in Bangladesh, representing national government, civil society, academia, and non-governmental organizations, identified through snowball sampling. The key informants were asked to describe the changing landscape of health care in Bangladesh from 1980 to 2015, with a particular focus on prevention and control of childhood diarrhoea and the involvement of non-governmental/private organizations in diarrhoea prevention and control programmes.

**Figure 1:**
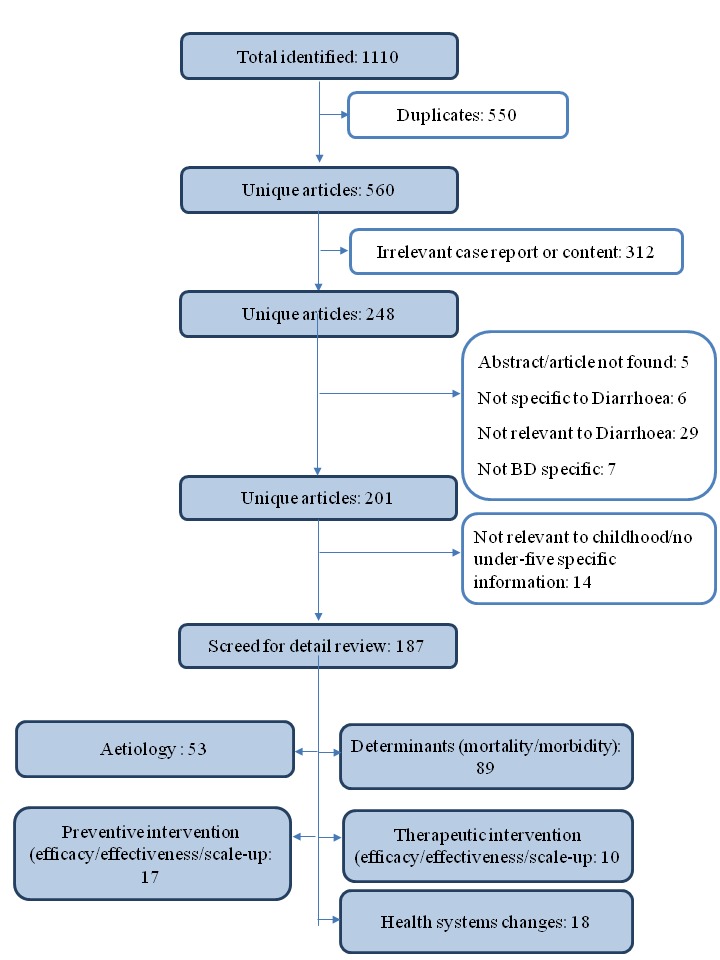
Article review diagram.

**Figure 2:**
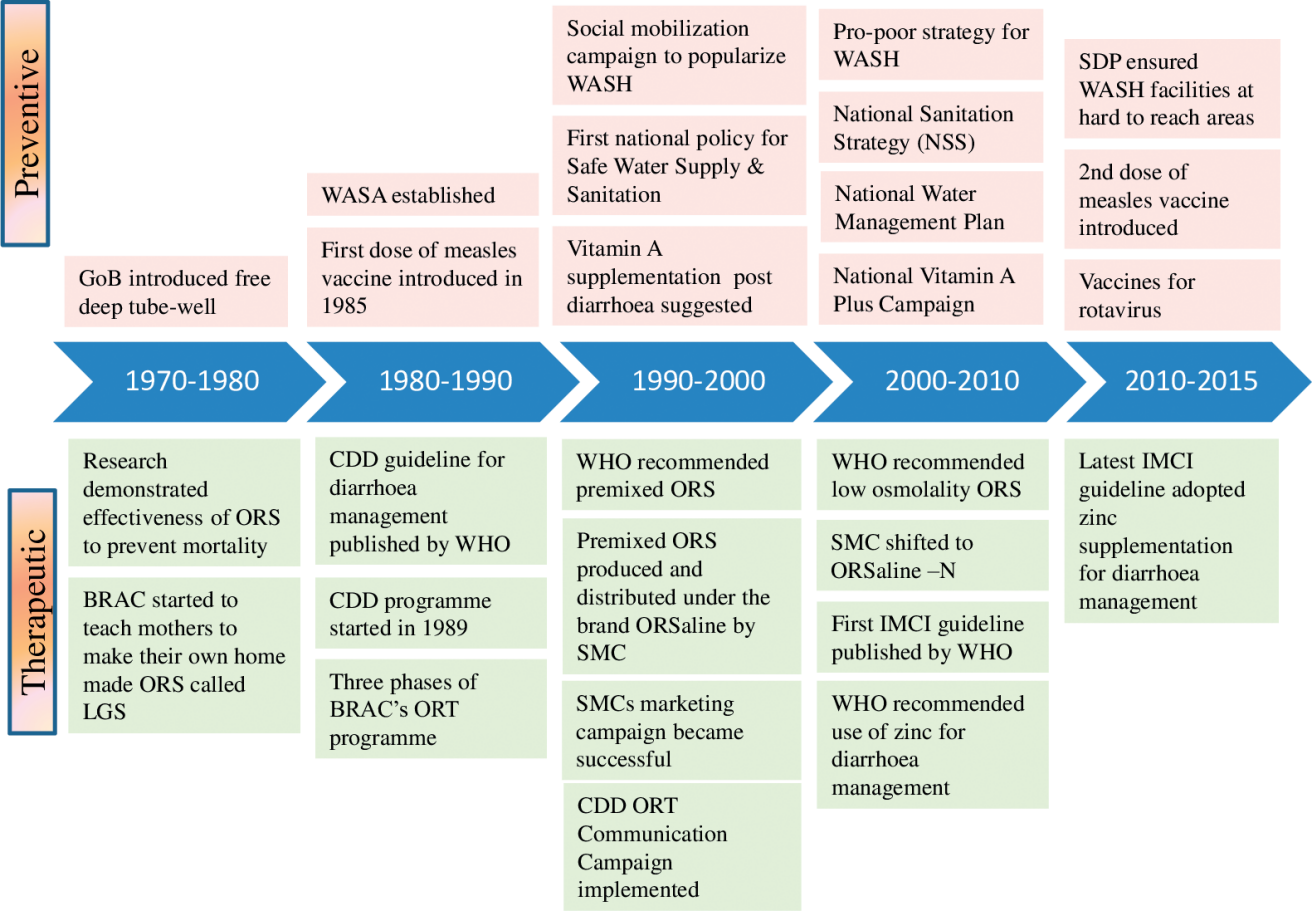
Key changes in policies and programmes for reduction of diarrhoeal diseases. GoB – Government of Bangladesh, ORS – oral rehydration saline, LGS – Lobon-Gur solution, WASA – Water Supply & Sewage Authority, CDD – control of diarrhoeal diseases, WASH – water sanitation and hygiene, WHO – World Health Organization, SMC – social marketing company, IMCI – Integrated Management of Childhood Illness, SDP – sector development plan

Findings from the key informant interviews were supplemented by in-depth literature review of published and unpublished documents from preceding three decades on direct diarrhoea management interventions and changes in nutritional status. Relevant publications between 1980 and 2016 were retrieved using the search terms ‘diarrhoea’, ‘infantile diarrhoea’, ‘morbidity'’, ‘disease incidence’, ‘case fatality rate’, ‘childhood mortality’, ‘newborn mortality’, and ‘Bangladesh’ in Pubmed, Embase, Scopus, and Global Health Ovid. As shown in **Figure 1**, a total of 1110 papers were identified that met the search criteria. After excluding duplicate articles, studies not specific to Bangladesh, non-specific case reports, and publications not specific to childhood diarrhoea, 187 articles were left. These papers were then reviewed to develop a comprehensive understanding of the socio-economic context and policy practices that underpinned the development and deployment of interventions for diarrhoea in Bangladesh. A diagrammatic summary of the health system and strategic changes that occurred in Bangladesh related to diarrhoeal disease management were built using these multiple sources of information (**Figure 2**).

### Statistical analysis

*LiST* was used to analyse and identify the drivers of diarrhoea mortality reduction, the proportion of mortality reduction and the number of lives saved attributable to each of the interventions. Assumptions and models used in *LiST* analysis to estimate diarrhoea mortality has been described in detail elsewhere [14]. This tool modelled effectiveness of interventions including changes in: i) nutritional status indicators (stunting and wasting), ii) coverage of diarrhoea management interventions (use of ORS and zinc for diarrhoea management, antibiotic for dysentery, and vaccination for rotavirus) iii) coverage of other preventive interventions (hand washing with soap, improved sanitation, safe disposal of excreta, improved water sources, vitamin A supplementation, age appropriate breastfeeding, and early initiation of breastfeeding) from 1980 to 2015. We also explored the contribution of drivers in two distinct time segments – 1980-2000 and 2000-2015 in the *LiST* analysis to see whether the effects of certain interventions on diarrhoeal disease mortality were different before and during the era of Millennium Development Goals (MDG). We also estimated the number of lives that can be saved by 2030 at near universal coverage (90%) of different interventions using 2015 as the baseline. We considered three scenarios for this analysis: i) direct diarrhoeal interventions, ii) direct diarrhoeal interventions and nutrition, iii) direct diarrhoeal interventions and nutrition drivers combined with WASH. The effectiveness values for the diarrhoeal interventions incorporated into the *LiST* tool have been published [14].

## RESULTS

### Trends in diarrhoea mortality and morbidity

**Figure 3:**
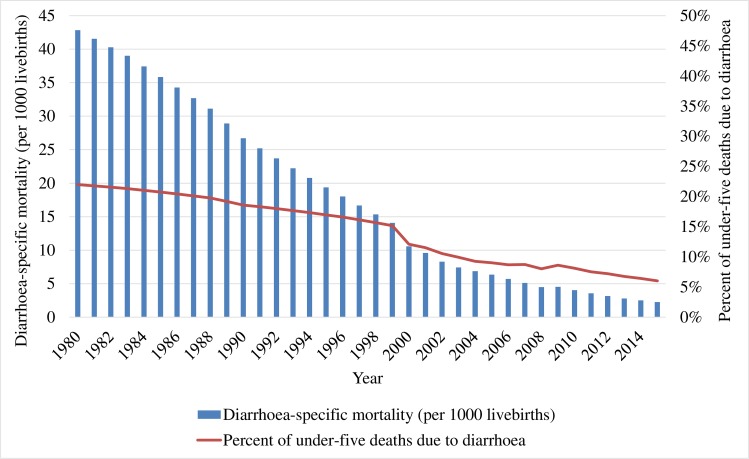
Trend in diarrhoea-specific mortality and deaths fractions among children under-five in Bangladesh between 1980 and 2015.

Bangladesh experienced a large drop in under-five mortality from 198.9 per 1000 live births to 37.6 per 1000 live births between 1980 and 2015 (**Figure 3**). Over the same period, diarrhoea mortality rates decreased among children under five from 15.1 per 1000 to 6.0 per 1000 live births [15]. Similar reductions were observed in the burden of diarrhoeal diseases. According to the BDHS from 1993 to 2014, the reported prevalence of diarrhoea in the previous two weeks among under-five children reduced from 12.6% to 5.7% (56% reduction) and the prevalence of diarrhoea with blood reduced from 2.8% to 1% (64% reduction) [7,11]. A hospital based study showed a decrease in the proportion of persistent diarrhoea from 8% in 1991 to 1% in 2010 [16]. The lack of nationally representative surveillance data limits a true estimation of the incidence of diarrhoea in Bangladesh, though we do have estimates from relatively smaller specific study populations at different time points [17].

The incidence of diarrhoea due to different aetiological agents varied over time. The major shift in aetiological agents has been from *Vibrio cholerae* and shigella to rotavirus [18]. The proportion of rotavirus diarrhoea increased from 25% in 1993 to 42% in 2012 among hospitalized patients [19]. Another study in seven urban hospitals in Bangladesh reported rotavirus infections in 64% of under-five children admitted for acute gastroenteritis during 2012-2015 [20]. A recent estimate based on a meta-analysis reported the incidence of rotavirus infections to be 100 cases per 1000 under-five children [21]. In contrast, a community-based study conducted in 2010-11 in the catchment area of six surveillance hospitals in Bangladesh estimated the incidence of severe diarrhoea due to cholera to range from 1 to 11 per 1000 among under-five children [22]. The Cholera Vaccine Investment Country Case Study in 2013 also reported a similar cholera incidence among under-five children, at a rate of about 8/1000 nationally and 11-12/1000 in the high risk districts [23].

### Historical and contextual drivers of child health

Bangladesh emerged as an independent, albeit impoverished, nation with a democratic government in 1971. In the nearly five decades since then, Bangladesh has experienced impressive economic growth, particularly in recent years. Per capita GDP increased from US$ 93 in 1972 to US$ 1210 in 2015 [24]. At the same time, adult female literacy rates in Bangladesh have increased to fairly high levels (from 18% in 1981 to 70% in 2016 [25]), testament to the success of three large-scale programmes started during early 1990s; food for education, and non-formal education by NGOs, and a female stipend programme [26]. Evidence suggest that gender equality and women’s empowerment in Bangladesh were positively related not only with economic development but also with improved nutrition and maternal and child health care practices and outcomes [27-29]. Working women were more receptive towards child immunization and the use of sanitary latrine facilities [30]. Sustained improvement in economic conditions, female education and women’s empowerment have resulted in improvements of maternal health and childhood nutrition indicators in last few years [27,31]. It is likely that economic growth, female education and women empowerment have also contributed to reduction of diarrhoea mortality in Bangladesh.

### Public sector health initiatives to combat diarrhoea deaths among children:

Bangladesh was an early adopter of programmes that aimed at preventing diarrhoea related deaths among children. It began in 1970 (when Bangladesh was still part of Pakistan) by the establishment of a Communicable Disease Control program to monitor diarrhoea epidemics in the country. After the Alma-Ata declaration in 1978, prevention and control of diarrhoeal diseases was adopted as a priority primary health care agenda in the health sector [32]. In recognition of the global burden of diarrhoeal diseases, WHO initiated the Control of Diarrhoeal Disease (CDD) Programme [33] which was later adopted by Bangladesh in 1989 [34] (**Figure 2**). In the late-90s, WHO and UNICEF launched the Integrated Management of Childhood Illnesses (IMCI) strategy to integrate management of common childhood illnesses including diarrhoea, pneumonia, malaria, measles and malnutrition [35]. The Government of Bangladesh, through the adoption of IMCI as an integrated strategy under the “Health and Population Sector Programme (1997-2002) (HPSP)” – the first Sector Wide Approach (SWAp), took a big step towards reducing under-five mortality. All the vertical programmes addressing common childhood illnesses were integrated into a single management guideline. Large investments made for the deployment of community health workers in the public sector as well as by NGOs, especially after Alma Ata declaration in 1978, helped achieve rapid uptake of community-based interventions like ORS for diarrhoea, family planning and immunisation [36].

### Therapeutic policies and programmes

During the 1960s, several studies were conducted at the Cholera Research Laboratory (now, the International Centre for Diarrhoeal Disease Research, Bangladesh; icddr,b) in Dhaka and the ground breaking ORS was formulated. This treatment proved effective in preventing diarrhoeal disease mortality among children [37]. From 1981, government started mass distribution of ORS through health facilities and during outbreaks under the National Oral Rehydration Project [38]. However, the scale-up efforts had limited success in the rural areas as there was no commercial production of ORS packets resulting in limited supplies [39]. During the same period, BRAC initiated a nationwide government of Bangladesh-approved programme to raise awareness of diarrhoeal illness and to teach caregivers on how to make *Lobon-Gur Solution – an easy-to-make home-made version of ORS* at home [37,40]. Despite a huge success in creating awareness on rehydration, *Lobon-Gur Solution* raised concerns of safety and reliability of the solution [39]. Consequently, the focus shifted to ORS packets, which was distributed with the brand name *ORSaline* by SMC (Social Marketing Company), the largest non-profit private social marketing company of Bangladesh. ORS was also distributed by government health facilities and community-based workers. In 2003, the WHO recommended a low osmolality ORS (ORSaline-N) for childhood diarrhoea management given its superior efficacy [41]. SMC switched to ORSaline-N and built a manufacturing plant for mass production and national distribution. Now, all public and private sector companies produce low osmolality ORS.

For almost a decade since 1989, the CDD programme in Bangladesh leveraged a government and non-government alliance and implemented several strategies and interventions to reduce diarroheal mortality and morbidity [34]. New insights gained from research at icddr,b and WHO training materials on interpersonal communication skills were incorporated in the training guidelines for health managers and providers [34]. The CDD programme established nine Diarrhoea Training Units (DTU) for active case management training to health care providers which achieved a high coverage among sub-district health managers and Medical Officers by 1997 [42]. This training brought improvement of knowledge and skills of the health workers which contributed by improving the quality of diarrhoea case management (**Figure 2**) [42].

**Figure 4:**
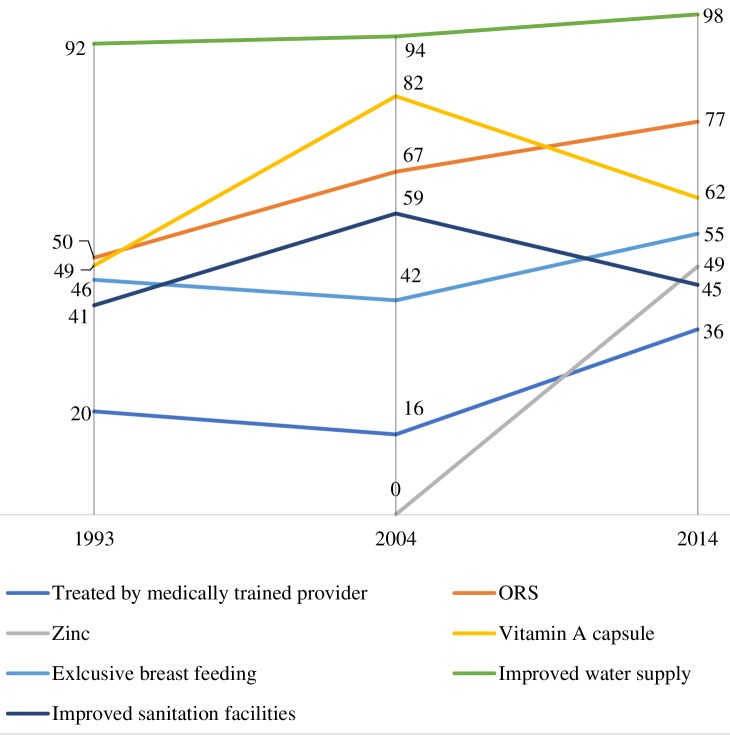
Changes in coverage (in percentage) of diarrhoea preventive and curative interventions between 1993 and 2014 (BDHS). ORS – oral rehydration saline.

Bangladesh has the world’s highest coverage of use ORS for diarrhoea [43]. Involvement of the non-government organizations (NGOs) and the private sector along with public sector stewardship was instrumental in popularizing ORS. Prior to the *Lobon-Gur Solution* initiative, the prevalent norm in the community was to restrict food and fluid to a child with diarrhoea [44]. With the *Lobon-Gur Solution* initiative, community health workers from BRAC provided door-to-door services to raise awareness of preventive and curative management of diarrhoea and care seeking form trained provider. This mass awareness raising effort helped substantially shift community norms from restricted feeding to feeding to the use of home-made solution for dehydration correction. An assessment in 1993 reported the success of the programme as 70% of the mothers could prepare *Lobon-Gur Solution* and they used oral rehydration therapy (ORT) (premix ORS or *Lobon-Gur Solution)* in more than 50% of diarrhoeal episodes among under-five children [40]. SMC promoted packaged ORS through national media campaign and ensured universal supply through outlets/shops in the community. Nonetheless, in 1996, CDD programme launched a large scale ORT communication campaign using a wide variety of communication channels to improve knowledge and awareness in the community on key rules of homecare – increased fluid, continued feeding and appropriate care-seeking during diarrhoea [34]. ORSaline became a common household commodity and the “go to” remedy for dehydration (**Figure 2**). The percentage of under-five children with an episode of diarrhoea receiving ORS increased from 50% in 1993 to 77% in 2014 (**Figure 4**) [7,11]. Bangladesh followed the WHO recommendation to use antibiotics only for dysentery and suspected cholera cases with severe dehydration. In addition, icddr,b in Bangladesh undertook pioneering research on the protective effect of therapeutic zinc during childhood diarrhoea on duration, incidence and mortality [3,38]. The research on the use of zinc supplements during diarrhoeal disease also found that it reduced the use of antibiotics without affecting ORS use [45]. Based on these findings, substantial national and international development efforts were directed towards scaling-up the use of zinc with ORS for the management of diarrhoea. Scaling Up Zinc for Young Children (SUZY) project was started in 2003 by icddr,b in collaboration with Government of Bangladesh [38,46]. WHO’s new recommendation on diarrhoea management in 2004 introduced the supplementation of zinc in addition to ORS ([47]). Zinc supplements were made available with the brand name of BabyZinc over-the-counter and rolled out in 2006 (38). In 2 years (by 2008), SUZY project increased awareness of zinc supplementation among caretakers to 50% in rural areas and 90% in urban non-slum areas [46]. The actual use of zinc during diarrhoea reached about 10% in rural regions and 25% in urban non-slum areas [46]. Since then, the coverage of zinc for diarrhoea management has been consistently increasing, reaching around 49% in 2014 (**Figure 4**) [11].

### Diarrhoea preventative policy and programmes

The Government of Bangladesh with support from UNICEF scaled installation of about 10 million deep tube-well (hand pump) free of cost in the 1970s to improve access to safe drinking water [48]. Improved sanitation became a priority during the mid-1990s. Several donor supported large-scale sanitation programmes with diverse intervention approaches, including provision of construction materials for improved sanitation facilities for free or on partial subsidies, promotion of hygiene practice through behaviour change communication (BCC) and creating enabling environment by destroying improper sanitation practices, were undertaken by both public and NGO sector initiatives between 1990 and 2014 [49]. To organize the Water, Sanitation and Hygiene (WASH) efforts, the first National Policy for Safe Water Supply & Sanitation (NPSWSS) was developed in 1998 followed by the National Sanitation Strategy in 2005 [50] (**Figure 2**). The involvement of the private sector in the production of consumer-oriented latrines and tube-wells at affordable prices increased the pace of the transition to safe drinking water consumption and community-led total sanitation. The National Sanitation Campaign 2003-06, integrating a successful blend of top-down and bottom-up approaches, was successful in achieving 100% latrine coverage in approximately 10 districts within a short time period [49]. The success of these programmes became evident by 2014, when almost every household in the country had access to safe drinking water along with 45% increase in access to not-shared sanitary latrines [51].

The measles vaccine was first introduced in Bangladesh in 1985 and was included in the routine Expanded Programme on Immunisation (EPI) schedule (**Figure 2**). The latest demographic and health survey reported 80% measles vaccination coverage by 12 months in 2014 [11]. Measles-rubella vaccine and a second dose of measles vaccine, which is protective against hospitalization for any infection [52], were introduced in late 2012. The rotavirus vaccine is commercially available in Bangladesh and is expected to be introduced in the EPI schedule in 2021.

Bangladesh started vitamin A supplementation of children in 1973 under the Nutritional Blindness Programme to address vitamin A deficiency [53]. Between 1973 and 1993, vitamin A capsules were distributed to children by frontline workers during household visits and during other service delivery contacts, achieving a coverage of 49% among children under three years of age [54]. However, the mass distribution of vitamin A capsules was adopted in 1995, linked with the national immunization days and also separately through vitamin A weeks [55]. In 2003, the GoB decided to observe the National Vitamin A Plus Campaign once a year; since 2004 National Vitamin A Plus Campaign has been organized twice a year (**Figure 2**). Coverage of Vitamin A supplementation in the last 6 months among under-five children peaked at 82% in 2004, and has since declined to 62% in 2014 (**Figure 4**) [9,11].

Since the 1980s, Bangladesh has adopted policies supporting breastfeeding and initiated breastfeeding promotion nationally. In 2003, the Ministry of Health and Family Welfare adopted a recommendation to breastfeed exclusively for the first 6 months of life. The National Infant and Young Child Feeding IYCF strategy was formulated in 2007 [56] and this was reflected in the 3^rd^ Health Population Nutrition Sector Development Programme 2011-2016 which prioritized growth monitoring and promotion, and counselling for infant and young child feeding, early initiation and exclusive breastfeeding [57]. Parallel to government efforts, NGOs have also played a crucial role in raising community awareness of IYCF practices. Bangladesh Integrated Nutrition Programme 1995-2002 and the follow on National Nutrition Programme partnered with NGOs for delivering direct nutrition interventions including promotion of IYCF practices [28]. Although the 3^rd^ Sector Programme mainstreamed delivery of nutrition services through the health system [58], several NGOs like BRAC, continued to promote IYCF practices by a large number of their own community volunteers. The multi-sectoral efforts to improve child feeding practices seem to have resulted in an increase in the prevalence of exclusive breastfeeding in the first six months of life from 46% in 1993 to 64% in 2011, though recent data shows a drop to 55% BDHS 2014 (**Figure 4**) [11].

### Interventions contributing to reduction of diarrhoea mortality – findings from LiST analysis:

We used *LiST* to estimate the impact of specific interventions on DSMR reduction based on the change in their coverage between 1980 and 2015 (**Table 1**). The *LiST* analysis was able to explain 95% of the reduction of DSMR during this 35-year period [15]. To examine if the scenario has changed in the MDG era, we also report impact estimates separately for the period before (1980-2000) and after 2000 (2000-2015). Impact was estimated by the number of lives saved and the percentage of DSMR reduction in under-five children attributable to each intervention for the overall period and two sub-periods (**Table 2**).

**Table 1:** Coverage/prevalence changes of interventions and risk factors from 1980 to 2015 in Bangladesh*

Factors/Interventions	1980 coverage (%)	2000 coverage (%)	2015 coverage (%)
Antibiotics for treatment of dysentery	4.0	8.2	9.7
Early initiation of breastfeeding	8.6	18.5	50.8
Hand washing with soap	3.1	12.7	17.0
Improved sanitation	41.4	45.4	60.6
Rotavirus vaccine: two doses	0.0	0.0	0.0
ORS – oral rehydration salt solution	0.0	62.6	77.0
Persistent diarrhoea treatment	0.0	0.0	33.0
Vitamin A supplementation	0.0	85.0	63.7
Zinc for treatment of diarrhoea	0	45.3	44.1
Childhood stunting (<-2 SD) rate	72.8	51.3	35.9
Childhood wasting (<-2 SD) rate	20.6	12.8	14.4
Exclusive breastfeeding <1 month	63	67.7	80.3
Exclusive breastfeeding 1-5 months	44.1	43	52.2
Any breastfeeding 6-11 months	98.1	97.4	96.4
Any breastfeeding 12-24 months	94.3	94.2	92.0

*Source: LiST analysis.

**Table 2:** Percentage of the diarrhoea specific under-five mortality reduction attributable to coverage of different factors in Bangladesh for the periods 1980-2015, 1980-2000 and 2000-2015 according to LiST modelling exercise*†

Factors/Intervention	2000 compared to 1980		2015 compared to 2000		2015 compared to 1980	
**Lives Saved**	**Reduction attributable (%)**	**Lives Saved**	**Reduction attributable (%)**	**Lives Saved**	**Reduction attributable (%)**
Changes in stunting prevalence§	23 969	21.5	7353	36.5	39 042	34.6
ORS‡	43 904	39.3	6230	30.9	31 485	27.9
Changes in wasting prevalence§	24 294	21.8	-	0.0	11 249	10.0
Vitamin A supplementation§	13 080	11.7	-	0.0	8124	7.2
Improved sanitation‖	1920	1.7	1782	8.8	7421	6.6
Zinc for treatment of diarrhoea‡	-	0.0	2448	12.2	5756	5.1
Hand washing with soap‖	3804	3.4	424	2.1	4446	3.9
Persistent diarrhoea treatment‡	-	0.0	1401	7.0	3295	2.9
Changes in age-appropriate breastfeeding practices§	-	0.0	451	2.2	1300	1.2
Antibiotics for dysentery‡	437	0.4	38	0.2	341	0.3
Early initiation of breastfeeding§	206	0.2	21	0.1	313	0.3
Rotavirus vaccine‡	-	0.0	-	0.0	-	0.0
Total	111 614	100.0	20148	100.0	112 772	100.0

*Lives saved and percent attributable in each refers to the change in a single year relative to a baseline year. Results are not cumulative over the time period.

†Source: LiST analysis.

‡Direct diarrhoea interventions.

§Nutrition interventions.

‖WASH interventions.

The major factors associated with the decline in DSMR in under-five children were stunting prevalence, ORS use, wasting prevalence, sanitation, vitamin-A and zinc supplementation, hand washing with soap and treatment of persistent diarrhoea. ORS use and reduction in stunting were the two key factors, with a combined impact of 63% or saving nearly 70 000 lives in 2015 compared to 1980. ORS had a higher contribution to DSMR reduction before 2000 with nearly 44 000 lives saved (39%) than reduction in stunting, which contributed 23 969 lives saved (22%). However, stunting had a relatively greater impact than ORS in the MDG era, 2000-2015, saving almost a thousand lives more than ORS. Zinc supplementation for the treatment of diarrhoea reduced DSMR by 12% during 2000 to 2015. Vitamin A supplementation contributed to DSMR reduction by 12% during 1980 to 2000, however, had no impact to the further reduction in diarrhoeal mortality between 2000 and 2015 due to stagnant coverage.

To assess potential impact of universal coverage (90%) of different interventions, we estimated the number of lives that can be saved in 2030 in three different scale-up scenarios (**Table 3**). Scenario 1 depicts the impact of five direct diarrhoea interventions only. Scenario 2 represents the effects of scaling up of the direct diarrhoea and nutrition interventions. Scenario 3 combines interventions in scenario 2 with WASH. The projections suggest direct diarrhoea interventions at 90% coverage will save an additional 3593 deaths in 2030 (Scenario 1). ORS had the largest contribution to diarrhoea management in this scenario (42%). When nutrition drivers are coupled with direct diarrhoeal interventions (Scenario 2), an additional 1410 lives will be saved in 2030. In this scenario, reduction in stunting prevalence appears to be the biggest contributor (21%) followed by ORS (17.5%). Nonetheless, the most substantial gains in mortality reduction could be achieved if both nutrition and WASH interventions are combined with direct diarrhoea interventions at 90% coverage, saving 5356 lives in 2030 (Scenario 3). Most of the additional lives saved in this scenario were attributable to handwashing with soap (18%) followed by reduction in stunting prevalence (17%).

**Table 3:** Attribution of reduction in mortality by 2030 to changes in intervention coverage and prevalence of risk factors for three scale-up scenarios in Bangladesh*,†

Factors/Intervention	Direct diarrhoea interventions (Scenario 1)		Direct diarrhoea interventions and nutrition (Scenario 2)		Direct diarrhoea interventions, nutrition and WASH (Scenario 3)	
**No. of lives saved**	**Reduction attributable (%)**	**No. of lives saved**	**Reduction attributable (%)**	**No. of lives saved**	**Reduction attributable (%)**
ORS	1522	42.4	875	17.5	622	11.6
Persistent diarrhoea treatment	614	17.1	348	7.0	248	4.6
Zinc for treatment of diarrhoea	529	14.7	301	6.0	214	4.0
Rotavirus vaccine	528	14.7	462	9.2	392	7.3
Antibiotics for dysentery	400	11.1	225	4.5	161	3.0
Vitamin A supplementation	-	0.0	246	4.9	211	3.9
Early initiation of breastfeeding	-	0.0	16	0.3	15	0.3
Changes in age-appropriate breastfeeding practices	-	0.0	780	15.6	649	12.1
Changes in wasting prevalence	-	0.0	693	13.9	493	9.2
Changes in stunting prevalence	-	0.0	1057	21.1	899	16.8
Hand washing with soap	-	0.0	-	0.0	967	18.1
Improved sanitation	-	0.0	-	0.0	485	9.1
Total	3593	100.0	5003	100.0	5356	100.0

*Each scenario represents scaling up to universal coverage of different intervention packages. Lives saved and percent attributable in each refers to the change in a single year relative to a baseline year. Results are not cumulative over the time period.

†Source: LiST analysis.

## DISCUSSION

Bangladesh has set an example for low and middle-income countries in the reduction of under-five child deaths due to diarrhoea. Our paper identifies the key factors contributing to this reduction in diarrhoea related under-five mortality. Improvement of nutritional status and achieving high coverage of ORS and zinc for the management of diarrhoeal episodes had the largest effects, although vitamin A also contributed along with smaller effects from most of the other interventions. Health system changes through timely adoption of evidence-based policies and programmes, effective collaboration with the private sector, NGO and social marketing efforts, and overall socio-economic progress also helped make this possible.

Introduction of ORS for the management of diarrhoeal episodes have consistently contributed to a large portion of the reduction in diarrhoea mortality in children in Bangladesh. Under strong government stewardship, robust efforts by the NGOs, especially BRAC, and the Social Marketing Company contributed to the success in the uptake of ORS for diarrhoea management by raising community awareness and ensuring the availability of ORS at an affordable price and easy access. Though an effective treatment, the coverage of using zinc to manage diarrhoea is not as high as ORS, it is still among the highest in the world [59,60]. The higher cost of zinc tablets when purchased from the commercial market and availability of zinc tablets free of cost only at public health facilities (which are not the dominant source of ORS) are key bottlenecks. This may explain the limited impact that zinc have had between 2000 and 2015. Training and involving informal providers for use of zinc for management of diarrhoea might be required to increase coverage. Additionally, introduction of rotavirus vaccine has the potential to further reduce diarrhoea related morbidities and death, as rotavirus is becoming a dominant aetiology in cases of severe diarrhoea [19].

Improvements in water sources and sanitation have contributed to approximately 10% reduction of diarrhoea related deaths between 2000 and 2015. Its impact was larger from 2000-2015 compared to the 1980-2000 period. This may be explained by Bangladesh’s development and adoption of a pro-poor strategy for water and sanitation in 2005 and a mass scale implementation of the proven interventions. Access to improved sanitation and hand washing with soap has improved from 41% to 61% and 3% to 17% respectively between 1980 and 2015 (**Table 1**). However, improvement in hand hygiene practices remains an unresolved agenda for Bangladesh. Data from hospital based studies among cholera patients in Bangladesh reported that only 4% (4/103) of cholera patients and their family household members had followed all key steps hand washing with soap [61]. Promotion of hand hygiene messages highlighting nutritional benefits has been recommended as the best motivator for development of hand hygiene practices [62].

**Figure 5:**
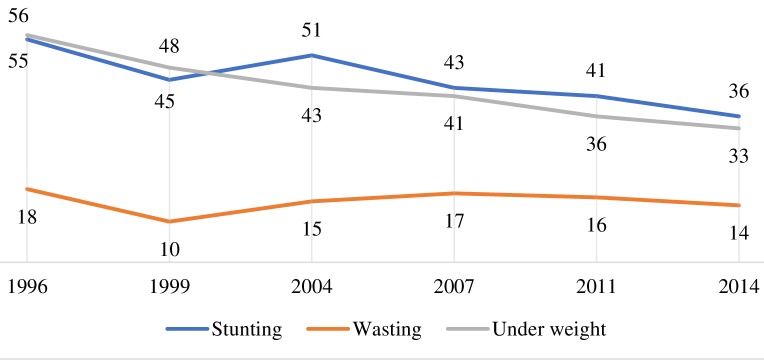
Changes in nutritional status (in percentage) among under-five children between 1996 and 2014 (BDHS).

Undernutrition has been often found to be associated with enteric infections leading to severe or persistent diarrhoea and associated deaths [63]. Bangladesh observed significant improvements in the reduction of childhood undernutrition over the past decades; stunting among under-five children declined from 55% in 1996 to 36% in 2014 (**Figure 5**). Results from the LiST analysis show the reduction in stunting as having the greatest contribution to diarrhoea mortality reduction among under-five children between 1980 and 2015. Recent evidence suggest preventing a child’s growth faltering would reduce the incidence of diarrhoea and associated adverse consequences including deaths [64]. Progress in nutrition in Bangladesh is mainly explained by the pro-poor economic growth, improvements in agricultural production and diversification, prioritizing nutrition sensitive interventions in policy, implementation of vertical programmes improving community awareness of women's rights and empowerment issues, response to nutritional emergencies, support from the NGO sector support in nutrition service delivery [28]. The reduction of DSMR could also partly be contributed by the major aetiological shift in causative agents of diarrhoea from Vibrio cholerae and shigella to rotavirus during this period. We could not take this into account in the LiST analysis due to inacessibility of consistent data and limitation of the tool.

The projection for the number of lives saved in 2030 based on only direct diarrhoea interventions were mostly the result of diarrhoeal management with ORS given that most childhood diarrhoeal disease deaths are due to acute watery diarrhoea [65]. Similarly, the proportional contributions of zinc treatment, rotavirus vaccine, antibiotics for dysentery and treatment of persistent diarrhoea on lives saved reflect their individual contribution in the context of Bangladesh. The significance of good nutrition is demonstrated in Scenario 2 (scaling up direct diarrhoea and nutrition interventions) where reduction of stunting prevalence made the most substantial contribution. Undernutrition was postulated to drive almost half of child deaths in low and middle-income countries, in the aggregate [66]. Stunting is a marker of chronic undernutrition and it stands to reason, based on previous studies and our projection scenario, that strategies that reduce prevalence of stunting can avert deaths due to diarrhoea further. Scenario 3 (scaling up direct diarrhoea and nutrition interventions and WASH) makes the case for basic sanitation like handwashing with soap.

Our study makes the case for policymakers to institutionalise deployment of evidence-based interventions at scale in collaboration with NGOs and the private sector to make large differences for population health over time. Massive declines in deaths due to diarrhoeal disease in Bangladesh happened because of the unique public-private partnership of the government with BRAC, SMC and other NGOs to mass produce and make available what works: ORS. This was after years of research by scientists to combine just the right amount of electrolytes with sugar to rehydrate children orally what previously could only be done by intravenous solutions by trained health professionals. ICDDR,B and BRAC played a pivotal complementary role to the public sector initiative in piloting the feasibility, effectiveness and subsequent mass deployment of this life saving solution.

## CONCLUSIONS

The clear success in diarrhoeal disease reduction in Bangladesh has made diarrhoea a forgotten agenda – with a general perception that it is no longer a priority issue. Intervention packages that optimise coverage of direct diarrhoeal interventions and promote nutrition and WASH strategies are needed to maintain the momentum of saving lives from a preventable and treatable illness like diarrhoea. Improvement in hand hygiene practices, increasing the coverage of zinc for the management of diarrhoea and continuing the progress in nutrition are some of the challenges that remain to further reduce the burden of illness and deaths from diarrhoea. Ongoing partnership with the NGO and private sector and utilization of pluralistic health system platforms for preventive and curative service delivery are needed to increase coverage of interventions and expedite the end of childhood diarrhoeal deaths in Bangladesh.
